# Indoor Air Bacterial Load and Antibiotic Susceptibility Pattern of Isolates in Operating Rooms and Surgical Wards at Jimma University Specialized Hospital, Southwest Ethiopia

**DOI:** 10.4314/ejhs.v21i1.69039

**Published:** 2011-03

**Authors:** Chalachew Genet, Gebre Kibru, Wondewosen Tsegaye

**Affiliations:** 1 School of Health Sciences, Samara University; 2 Department of Medical Laboratory Sciences and Pathology, Jimma University

**Keywords:** Indoor air, hospital environment, surgical site infection, Southwest Ethiopia

## Abstract

**Background:**

Surgical site infection is the second most common health care associated infection. One of the risk factors for such infection is bacterial contamination of operating rooms' and surgical wards' indoor air. In view of that, the microbiological quality of air can be considered as a mirror of the hygienic condition of these rooms. Thus, the objective of this study was to determine the bacterial load and antibiotic susceptibility pattern of isolates in operating rooms' and surgical wards' indoor air of Jimma University Specialized Hospital.

**Methods:**

A cross sectional study was conducted to measure indoor air microbial quality of operating rooms and surgical wards from October to January 2009/2010 on 108 indoor air samples collected in twelve rounds using purposive sampling technique by Settle Plate Method (Passive Air Sampling following 1/1/1 Schedule). Sample processing and antimicrobial susceptibility testing were done following standard bacteriological techniques. The data was analyzed using SPSS version 16 and interpreted according to scientifically determined baseline values initially suggested by Fisher.

**Results:**

The mean aerobic colony counts obtained in OR-1(46cfu/hr) and OR-2(28cfu/hr) was far beyond the set 5–8cfu/hr acceptable standards for passive room. Similarly the highest mean aerobic colony counts of 465cfu/hr and 461cfu/hr were observed in Female room-1 and room-2 respectively when compared to the acceptable range of 250–450cfu/hr. In this study only 3 isolates of S. pyogenes and 48 isolates of S. aureus were identified. Over 66% of S. aureus was identified in Critical Zone of Operating rooms. All isolates of S. aureus showed 100% and 82.8% resistance to methicillin and ampicillin respectively.

**Conclusion:**

Higher degree of aerobic bacterial load was measured from operating rooms' and surgical wards' indoor air. Reducing foot trafficking, improving the ventilation system and routine cleaning has to be made to maintain the aerobic bacteria load with in optimal level.

## Introduction

Surgical site infection (SSI) is the second most common health care associated infection next to hospital acquired urinary tract infection (1). The prevalence of SSI varies from country to country depending on level of adherence to infection prevention practice measures in a given health care setting (2). The infection, which is an important clinical indicator for quality of patient care and infection control (3), is primarily determined by the overall contamination level of hospital environment like indoor air together with the surgeon's technique during the operation, patient's degree of susceptibility, insertion of foreign material or implants, appropriateness of surgical preparation, adequacy and timing of antimicrobial prophylaxis (4).

Thus to achieve acceptable performance, operating rooms (ORs) and surgical wards (SWs) should accomplish a complex range of infection control measures by considering different contamination risks for SSI because a well implemented infection control program can reduce the incidence of hospital acquired infections (HAIs) by around one-third (though eradication is impossible) (5) as it is done in countries like USA (6). One of the risk factor for the development of SSI is bacterial contamination of indoor air in ORs and SWs (7). So, in any hospital which performs different surgical procedures, the hospital ORs and SWs should be well designed interms of ventilation and air-conditioning (6, 8) because such environments are one of the settings which require the highest hygiene standards than other settings in there (9).

ORs' and SWs' indoor air (which places patients at a greater risk than the outside environment) could be polluted with bacterial pathogens released into it from various sources (10). Environmental surface reservoirs like floors, patients and carrier health personnel, construction activities and delayed maintenance can act as a source for microbiological air pollution through shedding and environmental disturbance during different activities (11, 12). Factors like number of visitors, extent of indoor traffic, time of day and the amount of materials brought in from outside aggravate the extent of air bacterial flora. In one study, for example, airborne dispersal of *S. aureus* is directly associated with the concentration of the bacterium in the anterior nares. Approximately 10% of healthy carriers will disseminate *S. aureus* into the air. Thus the microbiological quality of air can be considered as a mirror of the hygienic conditions of the operating room (12, 13, 14) since reduction of airborne bacteria in the operating room by about 13-fold, for example, would reduce the wound contamination by about 50% (15).

Most of the infections arising from indoor air could potentially be prevented through adequate application of infection control practices (16). For instance, measuring the degree of bacterial contamination of indoor air and the susceptibility pattern of the isolates to commonly used antibiotics in the area will help to select appropriate antibiotics for empirical therapy. This also helps to revise and, if necessary, design appropriate hospital infection prevention protocols in an effort to minimize the incidence of costly SSI. Moreover, it provides the tools needed to localize the source and control the spread of SSI (17). Therefore, this study was conducted to determine the degree of bacterial contamination of ORs and SWs indoor air with respect to acceptable bacterial load standards and measure antimicrobial susceptibility pattern of the isolates.

## Materials and Methods

The study was conducted from October to January 2009/2010 in Jimma University Specialized Hospital (JUSH), located 352 Km from Addis Ababa, south west Ethiopia. JUSH is the only hospital in the town with 300 beds and offers different specialized medical services for people living in the south west of the country. The general surgical department consists of two ORs and four SWs. About five major operations (both elective and emergency operations) are conducting on daily basis. During air sample collection on average 20 students were usually present in each operating room to attend the surgical procedures-even more students would be enrolled in each surgical ward in morning round sessions.

For the purpose of air sampling, locations in the operating rooms were divided in to three zones in accordance with working group of the Scottish quality assurance specialists interest group (18): Critical Zone (where actual surgical procedure is performed), Intermediate Zone (where surgeons, their assistants and students change their clothing), and non Critical Zone (where sterilization and packing of surgical equipments is performed). The SW units are comprised of two female rooms (FR-1 & FR-2) and two rooms (MR-1 & MR-2) for males with a total of 53 beds. Both FR-1 and MR-1 are adjacent rooms locate at the left side of operating theater is connected by long corridor with the other two adjacent rooms (FR-2 & MR-2) located at the right side of it. The air samples for the study were taken from the following five units of Surgery Department: Critical Zone (OR-1 & OR-2), Intermediate Zone which comprises SCS (surgical close store), FCR (female clothing room), and MCR (male clothing room), non Critical Zone which include SR (sterilizing room), PR (packing room), and SMS (sterilized material store), Female surgical wards (FSWs) and Male surgical wards (MSWs).

Accordingly a total of 108 (36 from ORs and 72 from SWs) indoor air samples were collected from these different units using Settle Plate or Passive Air Sampling method following 1/1/1 schedule (a nine cm in diameter sterile Petridish with 5% Sheep's blood agar was left open to the air for an hour, a meter above the floor and a meter from the wall) in OR and SW units (19). During air sampling sterile gloves, mouth masks and protective gown was worn to prevent self contamination of the 5% Sheep's blood agar plate (Oxoid, UK) and the agar plate was checked visually for any bacterial growth before it was used. The air samples were collected in 12 rounds (once per week in OR and SW units). In each round, the air samples in SWs were collected three times considering the most representative hours of the day (at 8–9 AM, at 11AM–12 PM and at 4–5 PM) within a given day. On the other hand, air samples in Critical Zone of ORs were collected during the ORs were both active and passive where as samples from Intermediate and Non-Critical Zone of ORs were collected in the morning and afternoon in each round.

Then, the air culture blood agar plates were transported to the microbiology laboratory and incubated aerobically for 24 hours at 37c°. The culture plates that show discrete macroscopic colonies were counted using plate colony counter. The colonies were assessed for the growth of potential pathogenic bacteria initially by colony characteristics, hemolysis pattern and microscopic examination of Gram stained smears. Then, these suspected colonies were sub cultured on MSA and 5% Sheep's blood agar (Oxoid, UK). And finally, further identification was done by carrying out catalase, Coagulase, mannitol fermentation, and bacitracin susceptibility tests following standard bacteriological techniques (20, 21).

The antimicrobial susceptibility testing was done on Mueller-Hinton agar (Oxoid, UK) for every potential pathogenic bacterial isolates with 13 antibiotics (Table 2) each by Kirby-Bauer disk diffusion method matching the test organism to 0.5 McFarland turbidity standards. Then, the susceptibility result was interpreted according to the principles established by Clinical and Laboratory Standards Institute (CLSI) by measuring the zone diameter of inhibition (22). Prior to application, antibiotic discs potency was checked by using S. *aureus* ATCC25923, *E. coli* ATCC 25922 and *P. aeruginosa* ATCC 27853 strains as control organisms. Finally, the data was processed for descriptive statistics using SPSS version 16 and Microsoft Excel and interpreted according to scientifically determined baseline values initially suggested by Fisher in the 1970s and now widely adopted by the European Cooperation for Accreditation of Laboratories (19, 23). The ethical clearance was also obtained from Jimma University ethical review board.

**Table 2:** Antibiotic resistance pattern of potential pathogenic bacterial isolates from indoor air of OR and SW units in JUSH; October–January 2009/2010.

						Antibiotics tested No (%) of resistance							
Organism													
	P	Amp	T	C	Ch	Met	Ox	Gm	Cf	Cro	Txs	E	Va
***S. aureus*** **(n=48)**	34 (71)	37 (77.1)	24 (50)	19 (39.6)	15 (31)	48 (100)	44 (91.7)	12 (25)	4 (8)	24 (50)	34 (71)	24 (50)	48 (100)
***S. pyogenes*** **(n=3)**	0 (0)	1 (33.3)	0 (0)	0 (0)	-	-	-	-	-	-	1 (33.3)	-	

## Results

The bacterial load of 108 indoor air samples (36 from ORs and 72 from SWs) showed wide range of variation. The highest mean aerobic colony counts of 465 and 461 colony forming unit (cfu)/hr were observed from FR-1 and FR-2 of SWs respectively. Increased bacterial load of 465cfu/hr and 461cfu/hr in OR-1 and OR-2 was also obtained in both operating rooms when they were passive. Similarly, the higher colony count of 92cfu/hr was observed in OR-2 when it was active and this was also beyond the acceptable limit indicated on the standard (Table-1).

**Table 1:** Total aerobic bacterial load of indoor air from SWs and Critical Zone of ORs against the standard in JUSH; October–January, 2009/2010

Site	Rooms sampled	Aerobic colony count/hr; No. (mean value)	Standard (cfu/hr) (9)*		
			Optimal	Acceptable	Unacceptable
**SW units**	FR-1	18 (465)	0–250	250–450	>450
	MR-1	18 (416)	0–250	250–450	>450
	FR-2	18 (461)	0–250	250–450	>450
	MR-2	18 (352)	0–250	250–450	>450
**OR units** **(Critical** **Zone)**	OR1-A	2 (43)	0–60	61–90	>91
	OR1-P	4 (46)	0–4	5–8	>9
	OR2-A	4 (92)	0–60	61–90	>91
	OR2-P	2 (28)	0–4	5–8	>9

(9)* reference number

FR-1: Female room-1, FR-2: Female room-2, OR1-A: OR1-active, MR-1: Male room-1, MR-2 Male room-2, OR2-A: OR2-active, OR1-P: OR1-passive, OR2-P: OR2-passive

**Figure 1:**
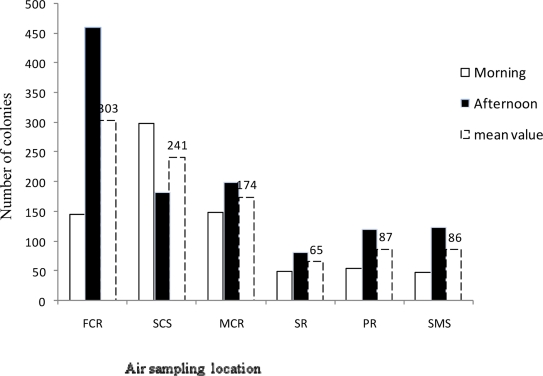
Total aerobic bacterial load of indoor air in Intermediate-Zone and Non-Critical Zone of ORs at JUSH, October–January 2009/2010. [Intermediate Zone (SR: Sterilizing room, PR: Packing room, SMS: Sterilized material store), Non-Critical Zone: (FCR: Female clothing room, MCR: Male clothing room, SCS: Sterilized cloth store)]

Both FCR (Intermediate Zone) and PR (Non-Critical Zone) of ORs also showed abnormally increased mean aerobic colony counts of 303cfu/hr and 87cfu/hr respectively. The colony counts in all ORs except Sterilized cloth store (SCS) were found to be higher in the afternoon than in the morning (Figure-1).

**Figure 2:**
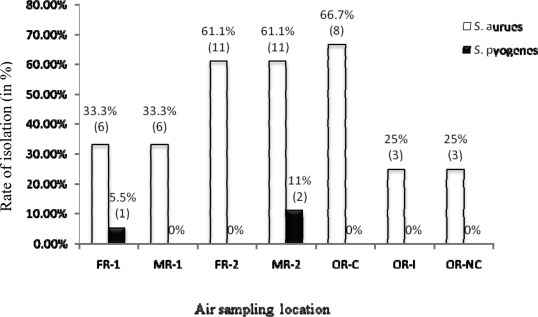
Isolation rates of potential pathogenic bacteria from indoor air in SWs and ORs at JUSH; October–January 2009/2010: % of isolation (No. of isolates) FR-1: Female room-1, FR-2: Female room-2, OR-C: OR-critical zone, MR-1: Male room-1 MR-2 Male room-2, OR-I: OR-intermediate zone, OR-NC: OR-non critical zone

Out of 108 air samples collected, only two bacterial species which are 3 isolates of *Streptococcus pyogenes* from SWs and 48 isolates of *Staphylococcus aureus* (70.8 % from SWs and 29.2 % from ORs) were identified. The isolation rate (66.7%) of *S. aureus* was significantly higher in Critical Zone than Intermediate and non Critical Zone of ORs (p<0.001). In addition, *S. aureus* was frequently isolated in FR-2 and MR-2 of SWs (Figure-2).

The susceptibility patterns of isolates revealed varying degrees of resistance to the antibiotics tested (Table-2). *S. aureus* showed 100% resistance to methicillin, 78% to ampicillin, 71.5% to penicillin and the least resistance which is 9.6% was observed for ciprofloxacin. On the other hand, *S. aureus* isolates were 100% sensitive for vancomycin (Table-2).

## Discussion

The microbiological quality of air can be considered as a mirror of the hygienic condition of the operating room (12–14). Observations of the number of bacteria carrying particles in air of surgical theatres and its premises may be required in safe working depends on the air content of bacteria being kept at a very low level. In hospital wards, in which there are outbreaks of cross infection, it may be also required to examine the air for its content of particular pathogens (32).

In this study the mean aerobic colony counts (ACC) of 46cfu/hr in OR-1 and 92cfu/hr in OR-2 obtained while both rooms were passive, were incomparably higher than the acceptable range of 5–8cfu/hr bacteriological standard set for passive rooms by Fisher *et al* (23) and Pasquarella *et al* (19). Similarly the mean ACC of 465cfu/hr observed in FR-1 and 661cfu/hr in FR-2 were beyond the recommended range of 250–450cfu/hr. The possible explanation for the increased load of mean aerobic counts could be the frequent human trafficking particularly students in these rooms as JUSH is a teaching and referral hospital in the region. Furthermore, the age and design of the hospital and its surrounding environments might also contribute for the current high aerobic bacterial load observed in these rooms.

The aerobic bacterial load in the OR's (Critical Zone) when they were active was 1.82 times higher compared with when they were at rest. This finding goes in line with the study conducted by Suzuki A *et al* (24) and also the standard by Fisher *et al* (23) which explained that the bacterial load of indoor air of OR is higher when it is active than when it is passive.

The mean ACC obtained in this study which is 463cfu/hr for FSW, 384cfu/hr for MSW and 58cfu/hr for OR (Critical Zone) (Table-1) were ten times greater than compared with a study conducted in Nigeria in 2007 by Oytayo VO *et al* that reported mean ACC of 42, 31 and 3cfu/hr for FSW, MSW and OR respectively (25). This observed variation in these two study results might include: (i) difference in representativeness of study subject where 108 indoor air samples analyzed in the present study compared to only 12 samples processed in the former study and (ii) differences in the number of persons present, the amount of their movements, and other dust raising activities in the rooms of these two hospitals. However, the high mean aerobic colony count observed in surgical department rooms of JUSH might be an indication of high level of environmental contamination.

Moreover, in this study the total indoor air aerobic bacterial load of Critical Zone was 58cfu whereas the load in the Intermediate Zone and non Critical Zone of ORs were 246 and 76cfu respectively (Figure 2). This finding goes in contrary to a study conducted by Suzuki A *et al* (1984) that the mean bacterial load of non Critical Zone was twice than in the Intermediate Zone (24). The high colony count observed in the Intermediate Zone when compared to non Critical Zone of ORs in this study might as a result of higher number of people (Staffs and students) were using it where as few people were working in the Non -critical Zone.

The bacterial profile of indoor air sample showed that *S. aureus* was the most frequently isolated species among potential pathogenic bacteria identified in both SW and OR units. The isolation rate of *S. aureus* was higher in Critical Zone (66.7 %) than Intermediate and non Critical Zones of ORs (each 25%). Similarly the isolation rate of *S. aureus* in Female room-2 and Male room-2 was twice than Female room-1 and Male room-1 of SW units (Figure 3). This variation may be due to difference in the presence of carriers (infected peoples) and in cleaning procedures that affect the load of *S. aureus* in the air as it was suggested by Chikere BC *et al* (26) and Suzuki A *et al* (11).

Furthermore, only 3 isolates of *S. pyogenes* and no Gram negative bacteria were identified from a total of 108 air samples examined. This might be due to the inability of Gram negative bacteria to survive for a long period in the aerosolized state as it was explained by Beggs BC (27) and to resist harsh conditions like drying (14, 28) as compared to their counter parts. This might be also true for low isolation rate of streptococci as these bacteria have relatively low survival time in the environment since they are fastidious and susceptible for environmental stress by their nature (29).

On the other hand, the drug susceptibility pattern of *S. aureus* which is 100% resistance to methicilin 82.8% ampcillin and 77.1% to penicillin obtained in this study is comparable with the study conducted by WoldeTenssay Z (31) where 93% resistance to ampicillin was reported.

In conclusion almost all indoor air samples except OR-1 when active and non-Critical Zone of ORs showed higher mean aerobic bacterial load compared to the standard (23). Thus, the bacterial load of indoor air was high which is considered as a risk factor for SSI since there is a linear relationship between bacteria count in air (operating rooms and surgical wards) and surgical site infection rate (20). In this study, *S. aureus* which is the most important cause of SSI (14) was the most frequently isolated bacteria with isolation rate of 38.9% and 47.2% in indoor air of ORs and SWs respectively. Thus, this is an indication that indoor air of ORs and SWs of JUSH has high risk of SSI for the patient and high risk of infection for the health personnel as well. Though the exact reason why the total aerobic colony count and frequency of potential pathogenic bacteria were high in the present study needs further research, there are different measures (30) which are known to reduce the total bacterial load and potential pathogenic bacteria to the optimal level. Therefore, foot traffic in and out of the ORs should be reduced, the ventilation system should be improved and appropriate educational intervention on routine cleaning practices should be provided for sanitary personnel.
